# Association between the Taq1A polymorphism and problematic media use in preadolescent children

**DOI:** 10.3389/fpsyg.2024.1395957

**Published:** 2025-01-08

**Authors:** Jennifer A. Emond, Timothy J. Renier, Dabin Yeum, Delaina D. Carlson, Grace A. Ballarino, Diane Gilbert-Diamond

**Affiliations:** ^1^Department of Biomedical Data Science, Geisel School of Medicine at Dartmouth College, Lebanon, NH, United States; ^2^Department of Pediatrics, Geisel School of Medicine at Dartmouth College, Lebanon, NH, United States; ^3^Department of Epidemiology, Geisel School of Medicine at Dartmouth College, Lebanon, NH, United States; ^4^Department of Medicine, Geisel School of Medicine at Dartmouth College, Lebanon, NH, United States

**Keywords:** problematic media use, Taq1A, screen time, children, addiction

## Abstract

**Objective:**

Problematic media use (PMU) is addiction-like media use. No study has examined if genetic factors for addiction relate to PMU during childhood. This study tested the association between genetic risk factors for addiction and PMU among 9-to-12-year-olds.

**Method:**

Data were from a cohort of 9-to-12-year-olds recruited from Northern New England, 2018–2022, for a study examining obesity risk among children. Two polymorphisms related to dopaminergic (*ANNK1* rs1800497 [the Taq1A polymorphism] and *COMT* rs4680) and one related to nicotinic (*CHRNA4* rs1044396) pathways that were previously associated with internet addiction or internet video game addiction in adolescents and young adults were genotyped. Parent-reported PMU for children was measured with a validated nine-item scale (range for final scores: 1 to 5); higher scores indicate more severe PMU.

**Results:**

Among children (*n* = 180; 43.9% female; 90.0% white, non-Hispanic; 82.2% of parents were college graduates), the median PMU score was 2.22 (IQR: 1.78, 2.78). In a linear regression model adjusted for child age, sex, European ancestry, and parent education, there was an additive association between the number of Taq1A1 alleles and PMU among children. Specifically, geometric mean PMU scores were 8.6% greater for each additional copy of the Taq1A1 allele (*p* = 0.030; *R*^2^ = 5.2%). No other polymorphisms were statistically associated with PMU at the *p* < 0.05 level.

**Conclusion:**

These preliminary findings suggest that a genetic predisposition to reduced dopamine sensitivity as indicated by the Taq1A polymorphism may relate to PMU in early adolescence. Findings need confirmation in larger samples.

## 1 Introduction

Problematic media use (PMU) reflects addiction-like media use that can negatively affect wellbeing. PMU includes preoccupation with media, use of media for mood regulation, increasing tolerance and withdrawal symptoms around media use, and media use interfering with family relationships (Domoff et al., 2019; Domoff et al., 2021; Kuss et al., 2014). Personal ownership of smartphones rapidly increases during the preadolescent years (The Common Sense Census: Media Use by Tweens and Teens, 2021), and PMU specific to smartphones has been documented in several studies of adolescents and young adults, with prevalence estimates ranging from 14 to 31% (Sohn et al., 2019).

The media content frequently accessed by preadolescents, including online video platforms and social media (The Common Sense Census: Media Use by Tweens and Teens, 2021), are highly engineered and persuasively designed to prolong engagement, with features such as intermittent reinforcement schedules for presenting highly enjoyable content and indicators of social approval (e.g., “likes”) (Franqueira et al., 2022; Griffiths, 2018; Kuss and Griffiths, 2017; Bringing Dark Patterns to Light: An FTC Workshop, 2022; Lindström et al., 2021). Importantly, a user’s interaction with online media is tailored using extremely sophisticated algorithms based on user-specific data to maximize the likelihood that the content presented is enticing (Bringing Dark Patterns to Light: An FTC Workshop, 2022). The design of online video platforms and social media have, in fact, been compared to slot machines (The Perfect Slot Machine, 2022), where rewards are anticipated but intermittent to prolong engagement (Lindström et al., 2021). Gaming apps, even those marketed as educational (Meyer et al., 2021), are also often persuasively designed with rewards that are “unlocked” with continued play. Online games popular with preadolescents also include virtual social networks that may increase a child’s affinity for mobile media use (Kuss and Griffiths, 2017). As presented in the Interaction of Person-Affect-Cognition-Execution (I-PACE) model for the development of internet use disorder (Brand et al., 2016), engaging with such rewarding content may train reactive and reward-seeking behavior that reinforces media use and may start a cycle of addiction-like behavior around media use. Preadolescents may be especially vulnerable to the persistent and highly accessible reward-based training that mobile media devices provide because neural networks that manage behavior and self-regulation do not fully mature until late adolescence (Strang et al., 2013; Klaczynski, 2004; Stevens, 2016). Importantly, such media use behaviors may be harmful to mental health. A meta-analysis of 18 studies that enrolled adolescents and young adults documented positive associations between problematic social media use and depression, anxiety, and stress (Shannon et al., 2022).

Few studies have investigated a potential genetic predisposition to addiction-like media use and all have been completed among adolescents or young adults with a focus on internet gaming disorders (Han et al., 2007; Lee et al., 2008; Montag et al., 2012; Jeong et al., 2017; Tereshchenko, 2023). Specifically, gene variants previously associated with internet gaming disorder or addiction include SNPs rs180097 (*ANKK1*) (Han et al., 2007), rs4680 (*COMT*) (Han et al., 2007), and rs1044396 (*CHRNA4*) (Montag et al., 2012; Jeong et al., 2017) and short/long variants of 5HTTLPR (Lee et al., 2008). In particular, each of these SNPs relates to addictive behaviors. rs1800497 (i.e., the Taq1A polymorphism) is in an exon of the *ANKK1* gene (previously believed to be within the *DRD2* gene) that impacts the dopamine receptor DRD2 (ANKK1, n.d.). The alternate allele (Taq1A1; a G > A variant) is associated with alcohol dependence (Smith et al., 2008) and other disorders of self-regulation including cocaine use (Blum et al., 1996) and overeating (Sun et al., 2017). *COMT* rs4680 results in a variant of catechol-O-methyltransferase (COMT), an enzyme that degrades catecholamines including dopamine (COMT, n.d.). The alternate allele (a G > A variant) results in greater instability and less enzymatic activity of COMT (COMT^L^) (Chen et al., 2004) and has been associated with the risk of major depressive disorder in some (Bækken et al., 2008; Wang et al., 2016) but not a majority (Antypa et al., 2013) of studies. rs4680 may also relate to nicotine dependence (Choi and Shin, 2015) including among adolescents (O’Loughlin et al., 2014), although findings are not conclusive (Choi and Shin, 2015). *CHRNA4* encodes the cholinergic receptor nicotinic alpha 4 subunit, a nicotinic acetylcholine receptor (CHRNA4, n.d.), and the alternate allele in rs1044396 (a G > A variant) has been related to less nicotine dependence (Feng et al., 2004). 5HTTLPR is a repeat polymorphism in the promotor region of *SLC6A4*, a gene that encodes the serotonin transporter (SLC6A4, n.d.). 5HTTLPR has two variants, a short and long version that differ in the number of 44-bp repeats in the promotor region (Heils et al., 2002), although extra-long variants have been reported (Goldman et al., 2010). The short-form variant relates to reduced serotonin transporter expression (SLC6A4, n.d.) and has been associated with an increased susceptibility to major depression and alcohol dependence (Oo et al., 2016), although findings are not conclusive (Goldman et al., 2010; Fratelli et al., 2020).

No studies have assessed if preadolescents with these genetic risk factors may be more susceptible to the rewarding effects of digital media and, in turn, be more prone to developing addiction-like media use behaviors. Therefore, the goal of this study was to leverage existing genetic data to examine the associations between previously documented genetic risk factors for internet addiction and problematic media use among 9-to-12-year-old children. It was hypothesized that children with SNPs reflective of a greater risk for addiction (e.g., the presence of the Taq1A1 allele in rs180097) or depression (e.g., variants in rs4680) would demonstrate greater PMU.

## 2 Materials and methods

### 2.1 Study sample

Data are from a cohort of 9-to-12-year-old children enrolled in a prospective study to analyze genetic risk factors related to pediatric obesity. Children and one parent each residing in Northern New England were recruited from the community. Data for this secondary analysis were collected from study activities that took place between April 2018 and April 2023 when saliva was collected for genetic analyses at the baseline visit. Children were eligible for the primary study if they were fluent in English. Children who had any relevant food allergies or dietary restrictions; were taking appetite- or attention-altering medications; had an attention, psychiatric, or neurological disorder; or who had a contradiction for an MRI scan (completed in the parent study) were excluded. This current analysis used data from the baseline visit that included the collection of a saliva sample from children for genotyping and an online survey completed by parents. Child–parent dyads were compensated up to $75 for completing that baseline study visit.

### 2.2 Genotyping

Saliva samples were collected from 189 children at the baseline visit with the Oragene Kit (DNA Genotek, Kanata, Canada), and per the original study protocol, isolated DNA was genotyped for more than 600,000 single nucleotide polymorphisms (SNPs) with the Illumina Global Screening Array 24 (v1.0 or v3.0); GenomeStudio software was used to generate clusters and genotype calls using manufacturer-recommended quality control thresholds (Illumina, 2014). As previously described (Renier et al., 2023), R (R Core Team, 2021) and PLINK (Chang et al., 2015; Meyer, 2023) were used for quality control. One participant was excluded for genotyping rate < 5%, five for outlying heterozygosity, and one for sex discordant from self-report. No sibling pairs existed in the sample (identity-by-descent pi-hat >0.2). Genotype data were available on 182 participants after quality control. SNPs deviating from Hardy–Weinberg equilibrium (Fisher’s exact test $p<1\times 10^{-6})$ , with more than 5% missingness, or with minor allele frequency < 1% were removed. Genetic European ancestry was determined as previously described (Renier et al., 2023; Meyer, 2023; Auton et al., 2015). To expand the scope of SNP data, haplotype-based imputation was performed using the Michigan Imputation Server (das et al., 2016) as previously described (Renier et al., 2023; McCarthy et al., 2016).

The current analysis initially considered four genetic variants previously related to internet video game addiction or internet addiction among adolescents or young adults: (Han et al., 2007; Lee et al., 2008; Montag et al., 2012; Jeong et al., 2017) the single nucleotide polymorphisms (SNPs) rs1800497, rs4680, and rs1044396 and the repeat polymorphism 5HTTLPR. SNPs rs1800497 (TaqA1 in *ANKK1*) and rs4680 (*COMT*) were directly genotyped and had complete information for all participants; rs1044396 (*CHRNA4*) was also directly genotyped but was treated as missing for three participants where genotype calling failed and imputation resulted in a posterior genotype probability below 0.9. The genotype frequencies for rs1800497, rs4680, and rs1044396 were each in Hardy–Weinberg equilibrium (exact test *p* = 0.847, 0.135, and 0.764, respectively). Short/long alleles of 5HTTLPR were assessed with two SNP haplotype proxies, including rs4251417 and rs2020934 (Wray et al., 2009), though the resulting genotypes deviated from Hardy–Weinberg equilibrium (exact test *p* = 0.013) and were excluded from downstream analyses.

### 2.3 Problematic media use

Parents completed the 9-item, Problematic Media Use Measure, short form (Domoff et al., 2019) for their child. This scale was developed to measure PMU among children 4-to-11-year-olds and was based on the nine DSM-V criteria for internet gaming disorder. Scale items address preoccupation with and unsuccessful control of media use, loss of interest in other activities, psychosocial and family-related problems because of media use, use of media to relieve mood, withdrawal, tolerance, and deception related to use. The internal reliability of the scale in the original validation study was high (*α* = 0.93) (Domoff et al., 2019). Parents rated how much each item reflects their child; all items are scored on a 5-point Likert scale anchored at never (1) and always (5). Final scores are the mean across all nine items with higher scores reflecting more severe PMU. There is no established threshold for this scale to create a binary classification of PMU, and scores were treated as continuous in all analyses. Two of the 182 participants with genetic data were missing the PMU measure, and thus, the final sample size was 180.

### 2.4 Covariates

Parents reported their child’s date of birth to compute age and their child’s biological sex, race, and ethnicity; parents reported their relationship to the child, their own educational level, annual household income, and marital status. Parents also reported which media devices their child used in the past month (TV set, desktop or laptop computer, touchscreen tablet, smartphone, video game console, etc.) and their child’s typical screen media use per week (hours) across all devices. Specifically, parents were asked to report the total time their child spent across all of those media devices for a weekday and separately, a weekend day. Parents were asked to include non-school leisure use only. Total leisure screen media use per week was a weighted sum of weekday (weight = 5/7) and weekend (weight = 2/7) use.

### 2.5 Statistical analysis

The distribution of PMU was right skewed and thus natural log transformed for regression analyses. Specifically, linear regression was used to model PMU (log-transformed) on each genetic risk factor, separately, adjusted for child age, biological sex, European ancestry, and parent education. Covariates were selected *a priori* and initially included household income; however, household income was not statistically associated with PMU and its inclusion did not improve the model fit as measured by the coefficient of determination (R^2^) and was thus not retained in the model. Regression analyses for each variant considered additive, dominant, and recessive models. The alternate allele of each SNP was modeled as the risk allele for each analysis. Residual plots were inspected and there were no apparent violations of model assumptions (normality of residuals, homoscedasticity, and no high leverage values per Cook’s distance). *p* < 0.05 was the threshold for statistical significance for all main effects. There were no adjustments for multiple comparisons. All analyses were conducted with the R Language and Environment for Statistical Computing, version 3.15.

### 2.6 Ethics statement

Written informed consent was collected from the parent, and children provided verbal and written study assent. All study activities were approved of by the Committee for the Protection of Human Subjects at Dartmouth College (Protocol 30723; approved 12/15/2017).

## 3 Results

**Figure 1:**
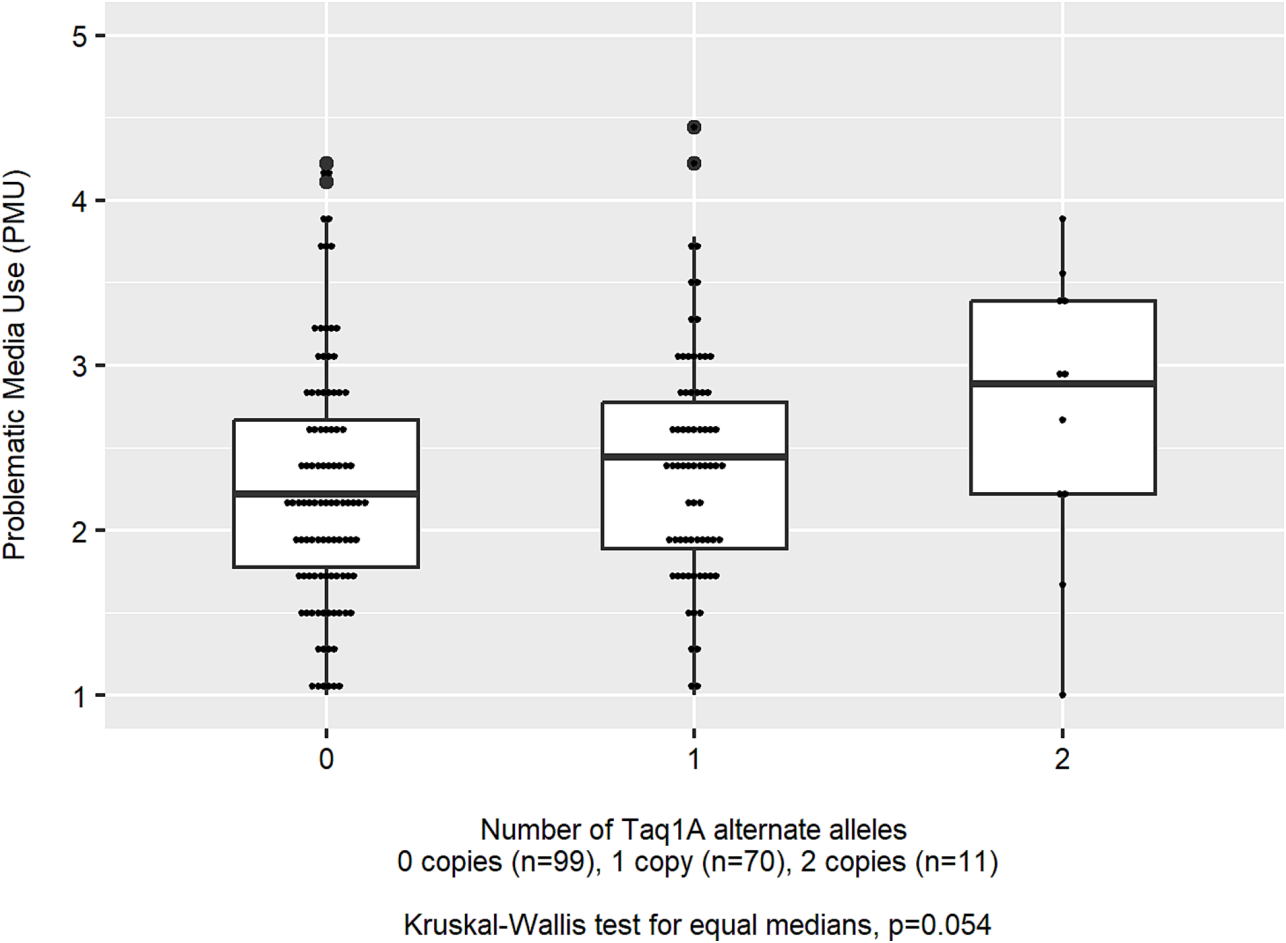
Distribution of problematic media use (PMU) scores by rs1800497 (Taq1A) genotype (*n* = 180).

The mean age of children in the analytic sample (*n* = 180) was 10.9 (SD 1.2) years, 43.9% of children were female, and most (90.0%) were identified by the parent as white, non-Hispanic. Families were largely from a higher socioeconomic status (Table 1). Children averaged 18.1 (SD 12.1) h per week of non-school-related screen media use, and nearly all (92.2%) of children used a touchscreen tablet or smartphone in the past month. The median PMU was 2.22 (IQR: 1.78, 2.78) which reflects a median response of rarely (2) to sometimes (3) across the nine scale items. Children’s leisure screen time per week was moderately correlated to PMU (Pearson’s r for log-transformed PMU and total screen time = 0.27; *p* < 0.001). The distribution of genotypes is presented in Table 2. Mean leisure screen media use per week was unrelated to rs1800497, rs4680, or rs1044396 genotype (all ANOVA *p*-values >0.117). Median PMU differed by rs1800497 genotype, which was of borderline statistical significance (Kruskal–Wallis *p* = 0.054; Figure 1) but did not differ by rs4680 (*p* = 0.917) or rs1044396 genotype (*p* = 0.944).

**Table 1:** Sample characteristics.

	*N* = 180
Child age, years, mean (SD)	10.9 (1.2)
Child biological sex,^a^ N (%)	
Male	101 (56.1%)
Female	79 (43.9%)
Child race and ethnicity, N (%)	
White, non-Hispanic	162 (90.0%)
European ancestry,^b^ N (%)	160 (88.9%)
Parent relationship to the child,^c^ N (%)	
Mother	148 (82.2%)
Father	25 (13.9%)
Other	5 (2.8%)
Not reported	2 (1.2%)
Parent’s educational level, N (%)	
High school graduate or less	22 (12.2%)
Associate’s degree	9 (5.0%)
Bachelor’s degree	49 (27.2%)
Graduate or professional school	100 (55.6%)
Annual household income (US $), N (%)	
<$25,0000	2 (1.1%)
$25,000-64,999	22 (12.2%)
$65,000-144,999	88 (48.9%)
$145,000-225,000	42 (23.3%)
>$225,000	18 (10.0%)
Not reported	8 (4.4%)
Parent marital status, N (%)	
Married or domestic partnership	146 (81.1%)
Child’s leisure media use	
Hours per week, mean (SD)	18.1 (12.1)
Hours per week, N (%)	
≤7	32 (17.8%)
>7 to 21	87 (48.3%)
>21	61 (33.9%)
Screen media device used in the past month, N (%)	
TV set	160 (88.9%)
Computer (desktop or laptop)	140 (77.8%)
Tablet or touchscreen device other than a smartphone	130 (72.2%)
Smartphone	110 (61.1%)
Touchscreen tablet and/or smartphone	166 (92.2%)
Video gaming system (handheld or console device)	79 (43.9%)
Child’s problematic media use, median (IQR)	2.22 (1.78, 2.78)

a Biological sex is reported for all children except one who reported gender preference.

b European ancestry defined by clustering of first two principal components of variation against known ancestry of samples from 1000 Genomes Project Phase 3 reference panel (see Meyer, 2023).

c Parent includes legal guardians and is inclusive of biological and non-biological parents.

**Table 2:** Genotype frequencies of the final three candidate genetic risk factors for problematic media use.

Genetic risk factor	Description	Homozygous referent allele	Heterozygous	Homozygous alternate allele
		N (%)	N (%)	N (%)
rs1800497	SNP in the *ANNK1* gene	99 (55.0%)	70 (38.9%)	11 (6.1%)
rs4680	SNP in the *COMT* gene	38 (21.1%)	101 (56.1%)	41 (22.8%)
rs1044396	SNP in the *CHRNA4* gene	41 (23.2%)	91 (51.4%)	45 (25.4%)

Among 180 9-to-12-year-olds recruited from the community. Alleles are reported in the forward orientation. Three participants are missing the rs1044396 genotype because genotyping results did not pass quality control checks. For each SNP listed, G is the reference allele and A is the alternate allele for European populations. See the dbSNP database from the National Library of Medicine, National Center for Biotechnology Information.

In adjusted regression models (Table 3), there was an additive association between the number of Taq1A1 alleles and greater PMU. Specifically, adjusted geometric mean PMU scores were 8.6% (95% CI: 0.8, 16.3%) greater for each additional copy of the Taq1A1 allele (*p* = 0.030; *R*^2^ = 5.2%) and were 2.13 for homozygous low-risk (*n* = 99), 2.31 for heterozygous (*n* = 70), and 2.58 for homozygous high-risk (*n* = 11) children. A dominant (*p* = 0.041; *R*^2^ = 4.9%) model was also supported by the data, although the additive model had a slightly better fit (Table 3). A recessive model was not statistically related to PMU (*p* = 0.209; *R*^2^ = 3.5%).

**Table 3:** Adjusted associations between the four candidate genetic risk factors and problematic media use, log-transformed.

Outcome: PMU, natural log transformed				
Genetic model specification				
Genetic risk factor	*N*	Additive	Dominant	Recessive
		b (95% CI)	b (95% CI)	b (95% CI)
rs1800497	180	0.086 (0.008, 0.163)^a^	0.100 (0.004, 0.195)^b^	0.128 (−0.072, 0.328)^c^
rs4680	180	−0.017 (−0.090, 0.055)	−0.045 (−0.163, 0.073)	−0.001 (−0.115, 0.113)
rs1044396	177	−0.006 (−0.074, 0.062)	0.003 (−0.111, 0.116)	−0.018 (−0.127, 0.091)

Among 180 9-to-12-year-olds recruited from the community. Three participants were missing the rs1044396 genotype because genotyping results did not pass quality control checks. Each model was adjusted for child age, sex, European ancestry, and parent education (as ordinal). Exponentiation of the beta coefficients minus one reflects the percentage increase in the geometric mean of PMU across each exposure level. For example, the geometric mean PMU was 11.6% greater for each additional copy of the high-risk Taq1A allele.

a *p = 0.030; Model’s adjusted R2 = 0.052.*

b *p = 0.041; Model’s adjusted R2 = 0.049.*

c *p = 0.209; Model’s adjusted R2 = 0.035.*

All other *p*-values for main effects ranged from *p* = 0.454 to *p* = 0.983.

No other allele variants were statistically associated with PMU at the *p* < 0.05 level. Findings were consistent when limiting the analysis to children of European descent, although the additive model for rs1800497 was not statistically significant (*p* = 0.097) and the dominant model for rs1800497 was borderline significant (*p* = 0.055) in this subset (Supplementary Table 1). In particular, *post-hoc* tests for an interaction between rs1800497 genotype and European ancestry were not statistically significant in the full dataset for the additive or dominant models (p-for-interaction = 0.273 and 0.858, respectively).

## 4 Discussion

In this preliminary study of 180 9-to-12-year-olds, rs1800497 (i.e., the Taq1A polymorphism) was positively associated with more severe PMU as measured with a validated scale appropriate for youth. This finding aligns with that of Han et al. (2007), where the rs1800497 alternate allele was more frequent among excessive internet video game players (*n* = 79) than age-matched controls (*n* = 75) in a study of male adolescents. This is the second study to suggest that rs1800497 relates to more addictive-like media use behaviors and the first to examine this association among preadolescents. Findings are important to understand the neurological mechanisms underlying the development of PMU and if some children may be more susceptible to PMU because of a genetic risk factor.

The alternate allele (A1) of rs1800497 results in a decreased density and binding potential of DRD2 in the striatum in humans and this association follows an additive model (Ritchie and Noble, 2003; Jönsson et al., 1999; Pohjalainen et al., 1998; Gluskin and Mickey, 2016). As stated by Blum et al. (1996) and Blum et al. (2000), a dampened responsivity to dopamine likely contributes to a reward deficiency syndrome that increases the risk of addictive, impulsive, and compulsive behaviors. The media contemporary preadolescents engage with is highly engineered to be persuasive and to prolong user engagement (Franqueira et al., 2022; Griffiths, 2018; Kuss and Griffiths, 2017; Bringing Dark Patterns to Light: An FTC Workshop, 2022; Lindström et al., 2021), which can result in compulsive use of mobile devices (Soror et al., 2015). Social media itself can be uniquely rewarding. Sherman et al. found heightened activity in reward networks of the brain when adolescents viewed photographs with many “likes” (Sherman et al., 2016) or when “liking” photographs from others (Sherman et al., 2018) using a simulated Instagram platform. Thus, it is feasible that those with reward deficiency syndrome may be most prone to develop PMU because of the rewarding nature of contemporary screen media. Unfortunately, our study did not measure children’s media use on the different device types or platforms, and we cannot test whether rs1800497 is related to the use of more (e.g., social media) vs. less (e.g., watching TV shows) persuasively engineered media.

This study did not find an association between PMU and rs4680 or rs1044396. rs4680 affects the stability of catechol-O-methyltransferase and the degradation of dopamine, suggesting that once introduced, dopamine is available in the synapses longer for those with COMT of low enzymatic activity (COMT^L^) (Chen et al., 2004). A previous case–control study of Asian male adolescents found a greater frequency of the allele encoding COMT^L^ among excessive internet game players (0.329) versus the control group (0.233) (Han et al., 2007). Regarding rs1044396, differing findings were reported in two previous studies examining rs1044396 and internet addiction, where the alternate (Montag et al., 2012) or reference (Jeong et al., 2017) allele related to greater internet addiction in two separate studies. This current study did not define cases with PMU and the overall mean value of PMU was low. It is possible that rs4680 or rs1044396 affects the severity or persistence of a media-based disorder once established but is not involved in the early development of that disorder. Conversely, the previous findings could have been confounded by comorbid mental health conditions (Lee et al., 2008; Ha et al., 2006).

Children’s total leisure screen media use per week was positively correlated with PMU in this current study. Screen time did not consider time spent on mobile devices specifically, and a direct test of persuasive media use and PMU is not possible in our study. However, 92.2% of preadolescents in the study used a mobile device in the past month, suggesting mobile device use was common. Screen time was unrelated to rs1044396 genotype, however, suggesting that the association with PMU is not fully dependent on total screen use quantity. Indeed, PMU captures many elements of problematic use including screen use to regulate negative mood. Longitudinal studies are needed to understand how a genetic risk for addiction relates to PMU considering the trajectories of screen use quantity and quality over time. That information is needed to best inform preventive interventions and guidance for parents and youth.

PMU was measured with a validated scale based on the nine DSM-V criteria for internet gaming disorder (Domoff et al., 2019). Importantly, this PMU measure is not specific to device type, which is an important consideration as preadolescent children frequently access online media via multiple devices including smartphones and touchscreen tablets (The Common Sense Census: Media Use by Tweens and Teens, 2021). However, measuring PMU in preadolescents is relatively new and thresholds to define clinically relevant PMU do not yet exist. Problematic smartphone use, defined as answering yes to at least six items of the 9-item Social Media Disorder Scale, was consistently related to lower mental, school, and social wellbeing in a study among 154,981 adolescents across 29 countries (Boer et al., 2020). That scale was also designed based on clinical criteria of addiction and scale items are similar to PMU items, suggesting that increased PMU may similarly relate to wellbeing among adolescents. The mean PMU was below 3 in this current study or a mean response of “sometimes” to each scale item. Additional studies are needed to understand how rs1800497 may affect PMU at more extreme levels, and if that genetic risk increases with age.

Strengths of this study include the collection of genetic data along with PMU among a sample of preadolescents. As a limitation, PMU and child media use were parent-reported and may be biased. This sample included children with lower levels of media use than national norms (The Common Sense Census: Media Use by Tweens and Teens, 2021), and the sample excluded children taking medications for attention disorders. Thus, included children were at a relatively low risk of developing more severe PMU. The sample was mostly white, non-Hispanic of higher socioeconomic status, limiting the generalizability of study findings. There were a limited number of preadolescents with two copies of the Taq1A1 allele (*n* = 11), which limits inference. The borderline findings among the subset of children with European ancestry may particularly be affected by a small sample size. We further did not measure comorbid mental health conditions that may confound reported associations. The goal of this study was not to conduct a genome-wide association study, but instead to test specific candidate SNPs as informed by the literature that suggests screen time may impact dopaminergic neural pathways.

## 5 Conclusion

In summary, this study found a positive, additive association between rs1800497, the Taq1A polymorphism, and more severe PMU among a sample of preadolescents. The effect size was small, and results need to be replicated in more diverse samples. Future studies should additionally include confirmation of PMU based on clinical criteria versus solely relying on parent reports Regardless, findings support that a neurological mechanism related to a reward deficiency may make some children more prone to developing addiction-like media use behaviors.

## Data Availability

The datasets presented in this study can be found in online repositories. The names of the repository/repositories and accession number(s) can be found at: https://www.ncbi.nlm.nih.gov/gap/, phs003550.v1.
