# Supplementary material for: Association between the Taq1A polymorphism and problematic media use in preadolescent children
Source: Front Psychol. 2025 Jan 8;15:1395957. doi: 10.3389/fpsyg.2024.1395957 (PMC11752389; doi:10.3389/fpsyg.2024.1395957)
Supplement: Supplementary file 1 [file Data_Sheet_1.docx]

**Supplemental Table 1.** Adjusted associations between the four candidate genetic risk factors and problematic media use, log-transformed, limited to children of European ancestry (n=160).

|  |  | **Outcome: PMU, natural log transformed** | | |
| --- | --- | --- | --- | --- |
|  |  | **Genetic Model Specification** | | |
| **Genetic risk factor** | **N** | **Additive** | **Dominant** | **Recessive** |
|  |  | **b (95% CI)** | **b (95% CI)** | **b (95% CI)** |
| **rs1800497** | 160 | **0.068 (-0.012, 0.149)^a^** | **0.096 (-0.002, 0.194)^b^** | 0.024 (-0.191, 0.239)^c^ |
| **rs4680** | 160 | 0.009 (-0.065, 0.083) | 0.026 (-0.097, 0.148) | -0.001 (-0.116, 0.115) |
| **rs1044396** | 159 | -0.009 (-0.080, 0.061) | -0.015 (-0.133, 0.103) | -0.011 (-0.122, 0.100) |

Among 180 9-to-12-year-olds recruited from the community, 160 of which were identified of European descent based on genetic information. One of these participants was missing rs1044396 genotype because genotyping results did not pass quality control checks. Each model was adjusted for child age, sex, and parent education (as ordinal). Exponentiation of the beta coefficients minus one reflects the percentage increase in the geometric mean of PMU across each exposure level. For example, geometric mean PMU was 10.0% greater for each additional copy of the high-risk Taq1A allele.
^a^P = 0.097; Model’s adjusted R^2^ = 0.062.
^b^P = 0.054; Model’s adjusted R^2^ = 0.069.
^c^P = 0.825; Model’s adjusted R^2^ = 0.046.
All other p-values for main effects ranged from p=0.682 to p=0.989.
